# The Relationship of Parental Pain Catastrophizing with Parents Reports of Children’s Anxiety, Depression, and Headache Severity

**Published:** 2018

**Authors:** Ghazale AKBARZADEH, Hojjat DANIALI, Mohsen JAVDZADH, Line CAES, Seyran RANJBAR, Mojtaba HABIBI

**Affiliations:** 1 Clinical Child and Adolescent Psychology, Shahid Beheshti University of Medical Sciences, Tehran, Iran.; 2 Department of Pediatric Neurology, Research Center, Research Institute for Children Health, Shahid Beheshti, University of Medical Sciences, Tehran, Iran.; 3 Faculty of Natural Science, Division of Psychology, University of Stirling, Scotland, UK.; 4 Department of Health Psychology, Tehran Institute of Psychiatry- School of Behavioral Sciences and Mental Health, Iran University of Medical Sciences, Tehran, Iran.

**Keywords:** Parental pain catastrophizing, Anxiety, Depression, Pain severity

## Abstract

**Objective:**

Parental pain catastrophizing is a construct recognized to have a significant impact on experience of pain in both children and parents. This research aimed to investigate the probable relationship of parental pain catastrophizing with the parent’s reports of children’s anxiety, depression and headache severity amongst Iranian parents of children with chronic or recurrent headache.

**Materials & Methods:**

This study was conducted in 2015-16, in two pediatric neurological centers located in Tehran, Iran; with a convenience sampling method and 212 parents (120 mothers and 92 fathers) of 132 children with a chronic or recurrent headache (migraine and tension-type). They completed the Pain Catastrophizing Scale; Numeric Pain Rating Scale, asking for the average of pain severity in last three months before the research, and the Anxiety and Depression subscales of the Children Behavioral Check List.

**Results:**

The mean age of parents was 35.41 yr (SD=5.58) and the mean age of children was 9.83 yr (SD=2.77). A total of 72 girls and 60 boys participated in this study with a mean pain severity for headache in last three months before the research of 4.99 (SD=2.63). Probable sex differences according to parental pain catastrophizing, pain severity, anxiety, and depression were assessed amongst parents. In the next step, the predictability of pain severity from parental pain catastrophizing was evaluated. Results indicated a significant relationship in maternal pain catastrophizing and estimates of pain intensity by mothers.

**Conclusion:**

These findings represent the importance of parent’s especially mother’s cognitive factors affecting their reports of their children’s pain and related emotional disturbances.

## Introduction

Chronic pain is a common, developmental problem that affects a vast number of children and adolescents (1, 2). Pain can interfere with children s’, and adolescent s’ daily functioning (3, 4) and increases the probability of developing a chronic disease in adulthood (5). Today, the amount of research focusing on family factors especially parents and its relationship to children’s pain is increasing (6). The experience of pain will capture our attention and that of others (7); others whose responses may in part affect the individual’s pain (8). As the children and adolescents are highly dependent on parents’ attention, this influence of others might be more important in children’s pain. Indeed, the parent’s behavior may have a profound impact on children and adolescent’s experience of pain (9, 10). For instance, parents’ behaviors could determine 55% of the variance in children’s behavioral disturbances (11). Certain parental behaviors (e.g. disappointing, over caring or overprotecting) are associated with long-term disturbances, difficulty in coping with pain, and increased risk of developing a chronic pain (12, 13). 

Moreover, family and parents are recognized as mediators of children’s adaptation to pain. In this regard, the term ‘encouraging the illness behavior’ about the concerning behaviors of parents confronted with children’s pain behaviors (which reflects behavior of over caring to child pain symptoms and encouraging children to abandon their daily activities) (12). To date, studies have shown a significant relationship between protective and concerning responses of parents and children’s pain outcomes (14). One of the significant factors in understanding interpersonal and psychosocial responses in child and parents in the realm of pain is the pattern of catastrophizing thinking. Catastrophizing thoughts about pain is defined as an exaggerated and negative orientation against actual or expected experiences of pain which include 1- rumination (tendency to focus on pain thoughts) 2- magnification (tendency to exaggerate extend of threat related to pain) 3- helplessness (tendency to take a helpless attitude in coping with experience of pain) (15). Catastrophizing factor initially was used in studying depression and anxiety (16) and main features of pain catastrophizing were similar with catastrophizing thoughts in depression and anxiety (17). Sullivan introduced this pattern of thinking to the domain of pain (18). Recently, researchers have also focused on parental pain catastrophizing about the child’s pain (19, 20). In response to child’s pain, catastrophizing parents use different behavioral patterns that sometimes may have considerable impacts on parent’s experience of child’s pain and the process of treatment and controlling the pain. For instance, parents who catastrophize about their children’s pain, when the child is in pain, they use more drugs to sedate the pain (21). There is a significant relationship between parental catastrophizing and increased child disability and pain severity (22, 23). Not only does catastrophizing relate to emotional disturbances of parents (24, 25), but it has also been associated with inappropriate responses of parents (26) and thus, might explain the deterioration of parental pain catastrophizing on experience of pain in children (24). Catastrophizing about child’s pain can be considered as a significant construct in understanding parents behaviours in conditions of acute and chronic pain in children (24), such as increased estimates and more acute feelings of parents disturbances such as depression (24, 25). This pattern of thinking can have a contribution in perception of pain in children. Parental pain catastrophizing and feelings of disturbances (emotional disturbances like anxiety and depression) is related to more impaired consequences like amplified disability (26) and more pain (27). In accordance with studies in recent two decades, catastrophizing thinking about pain can be considered as a vulnerability factor against chronicity of pain. In other words, this construct is not just related to the heightened levels of pain severity or emotional disturbances (depression or anxiety), but catastrophizing increases the likelihood of chronicity of pain for a longer period of time (28).

While there is magnificent work done in the realm of catastrophizing thinking of people in pain, little is discovered about the catastrophizing thoughts of ‘important others’ related to the person in pain. In particular, little is known about the extent to which parental catastrophic thinking influences how parents interpret the emotional responses of their child’s pain experiences. Consequently, the current study was set out to investigate the effects of parental pain catastrophizing on estimates of pain severity, depression and anxiety of children and therefore to study the impacts of parental pain catastrophizing on the pain-related reports of parents about children’s pain. Furthermore, most of the studies in this field have focused on mothers levels of pain catastrophizing, (24) and in this way, the generalizability of findings is limited (29) and there is lacking knowledge about the different impacts of fathers and mother’s pain catastrophizing (26). Using a sample of schoolchildren, one study showed that mother’s catastrophized more about their child’s pain than fathers (30). Similarly, there is a significant difference in parental pain catastrophizing between mothers and fathers (29). There is a difference in pain catastrophizing in men and women (31). However, cultural aspects could be at play and we need to ask whether Iranian fathers and mothers would also differ in levels of parental pain catastrophizing? Pain catastrophizing factor has a crucial role in explaining sex-related differences in clinical pains (17). 

The main aim of this research was to investigate the relationship of parental pain catastrophizing with estimates of pain severity, anxiety, and depression in a sample of parents of children suffering from chronic or recurrent headache and explore whether these associations are different in fathers and mothers. 

## Materials and Methods

**Participant**s

This descriptive cross-study used the same sample initially collected for investigating the psychometric properties of Persian version of PCS-P conducted in 2015-16, in two pediatric neurological centers ( Mofid Pediatric Hospital and Children Medical Center) located in Tehran, Iran; (32). Participants were 286 adult parents of 173 children suffering from chronic or recurrent headache (migraine or tension-type). This sample was gathered using a convenience sampling method.

Inclusion criteria entailed 1) the presentation in one of two centers, 2) child age between 7 and 16 yr; 3) an appropriate comprehension of Persian, 4) no underlying malignant disease; 5) having at least 7 yr of school education for parents, and 6) having headache (migraine or tension-type) for at least three months for children. Parents and the child interviewed with a pediatric neurologist and received a diagnosis of a headache (mostly migraine and tension-type). After that, one of the researchers introduced the project to parents and if they agreed to participate, checked the inclusion criteria with them. Out of this population, 21 children were removed from study because of having headache less than three months (counting for 35 parents), 7 children had another comorbid or underlying chronic disease (like cancer) (counting for 14 parents), and 8 children were younger than 7 yr old (counting for 15), and 10 parents were removed due to not meeting the criterion for school education years (counting for 5 children). This resulted in a sample of 212 parents which included 120 mothers (56% of whole sample) (M age=33.26 yr, SD=4.81,) and 92 fathers (M age=38.21 yr, SD=5.28) of 132 children (72 girls (54.5%) and 60 boys; Mage=9.83, SD=2.77). Overall, 75% of mothers were housekeeper and 50% of fathers were employees. The majority of the parents (84%) at least had a diploma or higher academic degree. The amount of sufficient sample size for achieving a multivariate regression is 15 subject per independent variable (33); as 3 independent variables in this research and 2 subscales for parental catastrophizing (rumination and magnification/helplessness), the number of 90 mothers and 90 father for each analysis was sufficient (33).

**Ethics**

This study was conducted between May 2015 and Sep 2016. It was approved by the Ethics Committee of Faculty of Psychology and Educational Sciences of Beheshti University, Tehran, Iran and by The Ethics Committee of Research Center of Pediatric Neurology of Mofid Hospital, Tehran, Iran as well.

**Procedures**

Two Iranian hospitals and a private clinic located in Tehran were selected for this study. Both of the hospitals were public. After children and their parents with headache complains, visited a pediatric neurologist and received a diagnosis of headache (migraine or tension-type), a trained clinical psychologist described the aims of the research. After checking their eligibility to participate in the study, they were asked for their informed consent to participate and their sociodemographic variables were recorded (age, sex, level of education). The participants were aware that their participation was voluntary, that the information collected was confidential, and that this information would be linked to a number alone and not to their name. 

The caregivers and the children were interviewed separately to avoid information bias. In participants who had their fathers accompanying them, researchers asked parent to answer the battery separately and without any consultation with each other. Eligible parent and children completed the questionnaire as part of their clinic visit. Researchers asked parents to complete the battery of questionaries’ about their experience with pain and about their thoughts about their children’s pain. The battery entailed the Persian Version of Parental Pain Catastrophizing Scale (PCS-P), Anxiety and Depression Subscales of Child Behavioral Check List (CBCL) and Numeric Pain Rating Scale (NPRS). 

**Measures**

***Parental Pain Catastrophizing Scale***

Parental catastrophizing was assessed with the Persian version of PCS-P (24). The English scale was translated into Persian following the guidelines of cross-cultural validation of self-report measures including translation and back-translation of the questionnaire (34). The PCS-P consists of 13 items describing different thoughts and feelings that parents may have when their child is in pain. Parents rate the extent to which they experience each of the thoughts and feelings using a 5-point scale (0=not at all, 4=extremely). The PCS-P yields a total score that can range from 0 to 52, and three subscale scores for rumination (‘‘When my child is in pain, I can’t keep it out of my mind”), magnification (‘‘When my child is in pain, I wonder whether something serious may happen”) and helplessness (‘‘When my child is in pain, there is nothing I can do to stop the pain”). In a sample of parents of children with chronic pain (N=107), an oblique factor-structure emerged to best fit the data in both parent samples (24). The total PCS-P score and the three subscales were all internally consistent with Cronbach’s alpha coefficients ranging from 0.81 to 0.93. In addition, criterion validity was demonstrated through significant relationship with child’s pain characteristics, parenting stress and parental emotional distress (24). The Persian version of the PCS-P (32) showed the best fit with two-construct factorial (rumination and magnification/helplessness) and this was in accordance with Persian version of PCS (35). Reported internal consistencies of total score, magnification/helplessness and rumination are orderly 0.89, 0.87 and .77 (36). Internal consistency of PCS-P in the current research for the total score was 0.89, and for two subscales were 0.85 and 0.77. 

***Numeric*** ***Pain*** ***Rating Scale (NPRS)*** **.** Is a straight line that in one of its end is zero (‘no pain’) and in the other is 10 (‘worst pain possible’). While using this scale, respondents are asked to choose a number between 0 and 10 declaring their pain intensity. Validity, reliability of this scale in clinical and research usage is confirmed (31). Some of the strengths of NPRS is being simple to utilize and scoring. In this research, by using the NPRS, parents were asked to rate the mean of the pain severity of their children’s headache in last three months before the research. 

***Child Behavior Check List (CBCL).*** The CBLC is aimed at children aged from 4-18 yr. This checklist is provided (37) and must be completed by a parent or a caregiver. The CBCL is built in two parts. The first part assesses competency inactivity, social and school. The second part includes 113 items about the specific disturbances and problems of children, and parents are asked to clarify their children’s condition in last 6 months for each item. This checklist has 8 factors: Anxiety-depressed, Withdrawn-depressed, Somatic complaints, social problems, thinking problems, attention problems, Rule-breaking, Aggressive behaviour and Other problems. Reported internal consistencies are acceptable (37). The psychometric Persian version of CBCL is investigated and the range of reported internal consistencies is acceptable (38). In this research, we used the items that assessed the anxiety and depression. Using these two subscales for children with chronic pain is previously done and is being accepted (39).

Parents also answered some researcher-made questions related to their socio-demographic information. 

**Statistical Analysis**

All data were entered and analyzed by SPSS software, ver. 22 (Chicago, IL, USA). After presenting the descriptive statistics and frequencies, we investigated the probable sex differences of parents in reported scales by using Multivariate Variance Analysis; and in the next step, using the Multivariate Stepwise Regression we investigated the relationship of mothers and fathers separately with estimates of pain severity, anxiety, and depression.

## Results

**Descriptive characteristics**

The average duration since the start of the headaches in children was 20.56 months (SD=19.22) and the mean of pain intensity in last three months was 4.99 (SD=2.63). The majority of children (74%) had headaches more than three times in last three months and 34% of these children experienced headaches more than three times per week. 

Table 1 presents the descriptive characteristics of the scales reported by parents separately for fathers and mothers. Means of mothers are higher in all of the scales (parental pain catastrophizing, anxiety, depression and pain intensity in last three months).

**Table 1:** Descriptive statistics of parents pain catastrophizing, pain severity, and reports of anxiety and depression

Scale	Sex	Mean	SD
Parental catastrophizing	Mothers	30.40	9.56
	Fathers	27.06	9.15
Child pain intensity	Mothers	5.57	2.67
	Fathers	4.60	2.39
Child anxiety	Mothers	4.54	2.73
	Fathers	4.51	2.46
Child depression	Mothers	3.98	3.16
	Fathers	3.78	3.11

*The mean of parental catastrophizing and reports of children pain intensity was higher in mothers, but there were not any considerable difference in the reports of anxiety and depression between mothers and fathers.*

*Parental catastrophizing: pain catastrophizing about children pain reported by mothers and fathers.*

*Child pain intensity: children pain headache intensity in the last three months before the research reported separately by mothers and fathers.*

*Child anxiety: the level of children anxiety reported separately by mothers and fathers.*

*Child depression: the level of children depression reported separately by mothers and fathers*

**Sex-related differences of parents in their reports**

Table 2 represents sex differences of parents in their reports of parental catastrophizing, pain intensity anxiety and depression using multivariate variance analysis. Accordingly, there was a significant difference in parental pain catastrophizing and report of pain severity between mothers and fathers. There was no considerable difference in mothers and fathers reports of children’s levels of depression and anxiety.

**Table 2:** Multivariate analysis for investigating the impacts of sex on catastrophizing, pain severity, anxiety and depression

Parameter	Multivariate variance analysis				Analysis of variance					
Dependent variable	Hotaling's amount	**F**	Degree of freedom	***P***	Source of change	Third type of sum of squares	Degree of freedom	**F**	***P***	Effect size
Catastrophizing	0.069	3.54	207 & 4	0.008	Between-group	582.01	1	6.60	0.011	0.030
					Within-group	18518.6	210			
					Total	19100.61	211			
Reported pain intensity					Between-group	48.62	1	7.42	0.007	0.034
					Within-group	1375.23	210			
					Total	1423.86	211			
Reported anxiety					Between-group	0.049	1	0.007	0.93	0.003
					Within-group	144.78	210			
					Total	1444.83	211			
Reported depression					Between-group	11.09	1	1.14	0.28	0.03
					Within-group	2034.92	210			
					Total	2046.01	211			

*According to this table and using multivariate analysis, there was a significant difference in parental pain catastrophizing and report of pain severity between mothers and fathers. There was no considerable difference in mothers and fathers reports of children’s levels of depression and anxiety in this study.*

*Catastrophizing: pain catastrophizing about children pain reported by mothers and fathers.*

*Reported pain intensity: children pain headache intensity in the last three months before the research reported separately by mothers and fathers.*

*Reported anxiety: the level of children anxiety reported separately by mothers and fathers.*

*Reported depression: the level of children depression reported separately by mothers and fathers.*

Table 3 represents the correlations between research variables. Mother’s catastrophizing about child’s pain was positively correlated with the reports of pain intensity, anxiety, and depression. With rise in mother’s pain catastrophizing, they reported higher levels of pain intensity, more anxiety, and depression about their children. Father’s catastrophizing about child’s pain was not significantly correlated with their reports of pain intensity and children’s level of depression. However, there was a significant correlation between father’s catastrophizing and their reports of children’s anxiety, which higher levels of catastrophizing related to higher report of child anxiety by fathers. 

**Table 3:** Correlational matrices for pain catastrophizing, pain intensity, anxiety and depression

Index	Sex	Catastrophizing	Pain intensity	Anxiety	Depression
Variable					
Catastrophizing	Fathers				
	Mothers				
Pain intensity	Fathers	0.20			
	Mothers	0.27**			
Anxiety	Fathers	0.25**	0.30**		
	Mothers	0.33**	0.26**		
Depression	Fathers	0.14	0.22*	0.57**	
	Mothers	0.20**	0.18*	0.59**	

*Catastrophizing: pain catastrophizing about children pain reported by mothers and fathers.*

*Reported pain intensity: children pain headache intensity in the last three months before the research reported separately by mothers and fathers.*

*Reported anxiety: the level of children anxiety reported separately by mothers and fathers.*

*Reported depression: the level of children depression reported separately by mothers and fathers.*

* *= 0.05,*

** *= 0.01. Mother’s catastrophizing about child’s pain was positively correlated with the reports of pain intensity, anxiety, and depression. Father’s catastrophizing about child’s pain was not significantly correlated with their reports of pain intensity and children’s level of depression. But there was a significant correlation between father’s catastrophizing and their reports of children’s anxiety.*

Within the multivariate regression analyses, maternal pain catastrophizing contributed significantly to the reports of pain intensity, suggesting that maternal catastrophizing accounted significantly for pain intensity reports, indicating higher catastrophizing was related to reports of more severe pains in last three months before the research (*β*=0.21, *t*=2.30, *P*<0.01) (Table 4). Results of Table 5 indicate that fathers catastrophizing has no contribution in reports of pain intensity of children’s headache. But father’s report of children’s anxiety accounted significantly for reports of pain intensity indicating higher anxiety of children related to more severe pain intensity reported by fathers (β=0.30, t=3, *P*<0.01) (Table 5).

**Table 4:** Regression analysis for investigating the impacts of maternal catastrophizing on reports of pain intensity

Dependent variable	Unstandardized coefficients		Standardized coefficients					Model summary				
	B	Standard error	Beta	t	sig	R	R^2^	Source of change	Sum of squares	Degree of freedom	F	sig
Constant	2.52	0.82	-	3.05	0.003	0.11	0.095 residual	regression	101.2	2	7.21	0.001
								820.388	117			
Mothers catastrophizing	0.063	0.027	0.21	2.30	0.002			total	921.59	119		
Report of anxiety	0.194	0.09	0.19	2.06	0.042							
Report of depression			0.043	0.39	0.69							

*Maternal pain catastrophizing contributed significantly to the reports of pain intensity, suggesting that maternal catastrophizing accounted significantly for pain intensity reports (β = 0.21, t = 2.30, P < .01). In the next step, mother’s reports of children anxiety had a significant contribution to the reports of children headache intensity. *

*Mothers catastrophizing: pain catastrophizing about children pain reported by mothers. *

*Reported pain intensity: children pain headache intensity in the last three months before the research reported by mothers. Reported anxiety: the level of children anxiety reported by mothers.*

*Reported depression: the level of children depression reported by mothers.*

**Table.5:** Regression analysis for investigating the impacts of fathers’ catastrophizing on reports of pain intensity

Dependent variable	Unstandardized coefficients		Standardized coefficients					Model summary				
	B	Standard error	Beta	t	Sig	R	R^2^	Source of change	Sum of squares	Degree of freedom	F	sig
Constant	3.28	0.50	-	6.55	0.001	0.30	0.091 residual	regression	47.68	1	9.05	0.003
								474.22	90			
Fathers catastrophizing			0.13	1.31	0.19			total	521.91	91		
Report of anxiety	0.29	0.09	0.30	3	0.003							
Report of depression			0.07	0.64	0.52							

*Father’s catastrophizing had no contribution in reports of pain intensity of children’s headache. reports of children’s anxiety accounted significantly for fathers reports of pain intensity (β = 0.30 , t = 3 , P < .01).*

*Father’s catastrophizing: pain catastrophizing about children pain reported by mothers. Reported pain Report of anxiety: the level of children anxiety reported by fathers.*

*Report of depression: the level of children depression reported by fathers.*

## Discussion

The present investigation deepens our knowledge of paternal catastrophizing and its influences on the experience of pain in children. The findings of the present study can be readily summarized: mothers’ level of catastrophizing was significantly higher than fathers. Of interest, mothers and fathers share similarities in catastrophizing, but also manifest some differences. Results of this study depict the significant relationship of maternal pain catastrophizing with the reports of pain intensity and emotional disturbances of children suffering from recurrent and chronic headache. Mothers, who used more catastrophizing thoughts about their children’s headaches, estimated more severe pain intensity about their children’s headache. There is a significant sex difference in this relationship, in which father’s catastrophizing had no contribution in the reports of pain intensity of children. These findings are in accordance with previous findings (29) that reported higher maternal pain catastrophizing than fathers. 

Another considerable point is that there was not any significant sex difference in reports of emotional disturbances by parents, indicating that mother’s and father’s reports of the children’s emotional disturbances are both considered in the same scope. Previous studies confirmed this sex difference in catastrophizing thinking (24, 29). These differences in maternal and paternal catastrophizing are important in understanding the child’s pain characteristics. Within the present sample, we understood that mothers’ but not fathers’ catastrophizing positively contributed to the child’s pain intensity reports. It is particularly rumination thoughts that may increase the attentional bias towards the pain experience (31). Therefore, and in line with previous studies indicating the contribution of attentional processes especially sustained attention in the impact of parental cognitive constructs affecting experience of pain in children, mothers who use more catastrophizing patterns of thinking, pay more attention to the child’s pain, and as a probable result convergent with operant model of chronic pain (40), the child’s perception of his/her pain intensity, might be reinforced and intensified as mothers pay more attention to the experience of pain in children (40). 

As a new aspect, this study showed how maternal pain catastrophizing could affect the pain-related reports of parents mostly used in clinical assessments. As in most of the clinical visits in pediatric clinics, this is mothers who report and clarify the complainants of children’s about their pain, this finding makes more sense and if the specialists were aware of the level of impacts of maternal catastrophizing on their reports of children’s pain, they might moderate this impact by providing more effective or educational therapeutic procedures. Another new aspect concerns fathers entailed in this study and were compared with mothers according to their pain catastrophizing level, reports of their children emotional disturbances and the intensity of pain intensity. At least in this study, father’s level of pain catastrophizing were not a proper predictive of the reports of their children pain intensity in comparison with mothers; this finding might be justified by the terms of more daily and hourly engagement of mothers to their children affairs, or the father’s ability to manage their thought procedures which lead to adjust the impacts of catastrophizing thinking on their reports. Probing each of these factors and their impacts in the field of children chronic and recurrent pain might be the task of further studies to be determined. 

There were some limitations in this study; first, all of the reports are obtained from parents and there are not any reports by children, further researches should cover this limitation. Investigating the impacts parental pain catastrophizing on children’s own reports of their pain intensity might enlighten new aspects of the interrelationship between parent's pain catastrophizing and their child perception of his/her experience of pain. Second, the small sample size of fathers obstructed researchers to have more analyses on father’s reports. Further researches should cover these limitations. The last limitation concerns the unspecified categorization of headaches in this research due to limited information children medical documents, which remains to be covered by further related studies.

**In conclusion, **the causal quality of these relationships and of the differences between mothers and fathers remains to be determined in future studies. Answers to these questions may help to develop interventions targeting maternal and paternal catastrophizing and then to evaluate the extent to which such interventions actually affect pain outcome in children. As a result, specifically tailored interventions for mothers and fathers could be developed. 
